# Influence of Kinesitherapy on Balance Reactions in Patients with Ischemic Stroke in the Chronic Period

**DOI:** 10.3889/oamjms.2015.105

**Published:** 2015-09-28

**Authors:** Danche Vasileva, Daniela Lubenova, Marija Mihova, Antoaneta Dimitrova, Kristin Grigorova-Petrova

**Affiliations:** 1 *University “Goce Delchev”, Faculty of Medical Sciences, Shtip, Republic of Macedonia*; 2 *National Sports Academy, Physical Therapy and Rehabilitation, Sofia, Bulgaria*; 3 *Ss Cyril and Methodius University of Skopje, Faculty of Computer Sciences and Engineering, Skopje, Republic of Macedonia*

**Keywords:** Kinesitherapy, Balance reactions, Ischemic stroke, Chronic period, Neurodevelopmental treatment, Physical activity

## Abstract

**AIM:**

The study aims to trace the influence of specialized kinesitherapeutic methodology (SKTM) on balance reactions in patients with ischemic stroke in the chronic period (ISChP).

**MATERIAL AND METHODS:**

A prospective, multicenter study with 56 patients with ISChP. Evaluation of balance reactions using Berg Balance Scale - BBS, includes implementation of 14 tasks with increasing difficulty reflecting the usual activities of everyday life. The first 5 assignments are used to assess the main balance potential and the remaining 9 (6th to 14th task) include more sophisticated balance tasks.

**RESULTS:**

The patients were found with a significant improvement in balance opportunities, according to the scale of Berg. Compared to initial data there is a significant increase in the number of points in the measured indicators for functional and static balance. In absolute terms, positive change is most pronounced during the 1st month with a level of significance of p <0.001.

**CONCLUSION:**

The applied specialized kinesitherapeutic methodology continued later as adapted exercise program at home, and significantly improved equilibrium reactions in patients with postural disorders because of ischemic stroke and is with a supportive prolonged exposure.

## Introduction

Stroke is the leading cause of disability, as approximately two thirds of patients have residual neurological damage, mainly hemiparesis, which affects balance and gait. Some of the major gait disturbances after stroke are characterized with inadequate postural control, higher risk of falling in all stages after stroke [1], walking slowly and a need of more energy in motion [2, 3].

Postural control is the ability to hold the centre of gravity of the body in the footprint while sitting and standing. It is a variable indicator that combines both mobility and stability and it is a necessary condition for taking and holding the necessary posture when performing controlled and coordinated motor activity [4]. Impaired postural control reduces the ability of independence in everyday life [5]. Patients who suffered stroke have limited physical activity that alters the extra sensorimotor control, postural control, musculoskeletal and autonomous control. This creates progressive cardiovascular risk for development of the disease, a predisposition to recurrent stroke, and delayed reactions [6-9], leading to long-term restrictions, hampered physical performance and social adaptation of the patients [10]. Asymmetric postural control and difficulty in walking in patients who suffered stroke are due to many factors, such as reduced muscle strength, imbalance in weight distribution, impaired proprioception, increased tendon reflexes, spasticity and impaired motor control [11] and it is obvious that the training and assessment are often focused on the tasks to pass the weight on the body [12-14].

With the development of neurorehabilitation and principles of motor control in recent years new therapeutic approaches have been developed for patients with hemiparesis. One is neurostimulilate therapy on Bobath (Neurodevelopmental Treatment - NDT) [15]. This approach is constantly evolving and widely used in neurological practice [16]. It is entirely based on the principles of motor training [17, 18]. These are related to stimulation of neuroplasticity and storage of the cells around the infarction zone. For example, neurons which are anatomically associated with the infarction zone, in the process of recovering from the depressing state, restore function by causing plastic changes like receptor hypersensitivity and dendritic growth of new internerval paths. Recent studies indicate that plasticity is further stimulated by physical activity [19, 20]. The central mechanisms of neuroplasticity are not fully understood. It is known that in the earlier period of brain injury processes of restitution, compensation and reorganization and adaptive strategies occur. In the late period (after 6 months) with functional imaging an organization of new neural network is established which covers the damaged premorbid network [21].

The aim of this study is to trace the influence of specialized kinesitherapeutic methodology (SKTM) on static and functional balance reactions in patients with ischemic stroke in the chronic period (ISChP), which was developed based on the principles of motor control, motor training and modern guidelines of neurostimulative therapy of Bobath (Neurodevelopmental Treatment - NDT).

## Material and Methods

### Methodology of the Study

This is a prospective, multicenter study with 56 patients with ISChP (32 men and 24 women with disease duration of 5 months to 1 year). The survey was conducted between 2012-2015 in a specialized office for physical therapy Faculty of Medical Sciences at the University “Goce Delchev” - Shtip, Macedonia and Specialized Hospital for post treatment and rehabilitation - Pancharevo - Sofia, Bulgaria.

The clinical characteristics of the patients are given in Table 1. According to the stage of Brunnstrom the severity of paresis is medium of the upper and lower limb. Patients can perform the following active movements: lifting the arm to 90 degrees, initial extension in elbow, wrist and fingers. In the lower limb possible movements are the following: extension in the knee and the initial dorsal flexion in the ankle joint. Muscle tone is slightly increased, according to the scale of Ashworth [22].

**Table 1:** Clinical characteristics of the contingent at the beginning of the study

Age	Weight	Height	Brunnstrom- upper limb	Brunnstrom - lower limb	Ashworth – upper limb	Ashworth – lower limb
X̄±S_D_	X̄±S_D_	X̄±S_D_	X̄±S_D_	X̄±S_D_	X̄±S_D_	X̄±S_D_
63.2±8.8	77.9±10.1	169.2±6.4	4.2±0.7	4.8±0.6	1.6±0.6	1.1±0.5

*X̄± SD – average value and standard deviation*.

Due to homogeneity in the study, patients were selected by the following criteria: not have severe respiratory insufficiency, cardiovascular insufficiency (third functional class), uncontrolled diabetes mellitus, cognitive and memory disorders, acute thrombophlebitis, severe decubital ulcer, severe orthopedic disorders impairing coordination and gait, ischemic heart disease, malignancies, severe progressive neurological disorders and to have given a written consent to participate in the study. All patients were able to move independently or with assistance and without serious problems in communication, with a pre-prescribed medication by neurologists, including antithrombotic and antihypertensive drugs.

For the assessment of the initial functional status of the patients, Brunnstrom test was used, whereas for measuring objectivity of muscle tone before treatment - the scale of Ashworth [22]. For determining changes in static and dynamic balance before and after the treatment the Berg Balance test was used. It has strong psychometric properties and it is valuable in the evaluation of clinical change balance after stroke [23]. The original test involves performing 14 tasks with increasing difficulty, reflecting the usual activities of everyday life (getting up from a sitting position, picking up an object from the floor, standing on one leg, turning, reaching, stepping on block). The first 5 assignments are used to assess the main equilibrium potential and the remaining 9 (6^th^ to 14^th^ task) include more sophisticated balance tasks. Patients’ abilities such as maintaining balance in performing tasks with gradual reduction of the footprint, weight transfer to the body, turning and reaching are assessed. The first task is performed in a sitting position and ends with standing on one leg. Assessment is carried out by using a 5 point scale (0-4) depending on the ability of performing a given task. The higher score means better ability for making that move. These grades are based on clearly defined criteria [24].

All indicators in the patients were evaluated four times - at the beginning of the study, on the 10th day, during the 1st month and during the 3rd month after the start of the SKTM.

### Methods of physical therapy

All patients were treated with antithrombotic drug and antihypertensive drugs and through a specialized 10-day SKTM, which later continues to be performed as adapted exercise program at home for a period of three months [15]. It was developed based on neurostimulative therapy of Bobath (Neurodevelopmental Treatment - NDT) and principles of motor control. It is applied daily of moderate exercise intensity and duration of about 40-50 minutes. In the introductory part, the exercises are aimed at preparing the body for the upcoming exercises, gradual adaptation of the cardiovascular system (chest and diaphragmatic breathing). The main part of the kinesitherapy includes exercises for the transition from the occipital lying to standing exercises for upper limb and control of the shoulder girdle, lower limb exercises and control of the body, pelvis and walking. The final part includes relaxation exercises to patients.

### Statistics

The obtained data were processed statistically using descriptive analysis, analysis of variance and alternative analyzes. Paired Samples Test is used to compare the parameters at the beginning, on 10^th^ day, during the 1st and during the 3^rd^ month after the kinesitherapy. Correlation analysis Pearson was used and p-value less than 0.01 were considered statistically significant.

## Results

**Figure 1:**
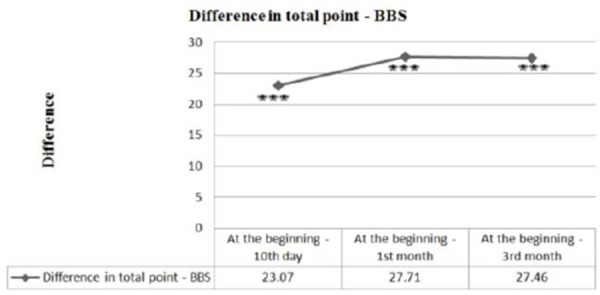
Change in the total number of points for static and functional balance, according to the scale of Berg presented as a ratio of the results and the initial (baseline) values; *** P <0.001 - a significant difference compared to the initial (baseline) values.

Results of the study in patients with ISChP before treatment and after kinesitherapy are summarized in Table 2 and Table 3, and the ratio between obtanied and baseline parameters and the importance of changes in the patients is shown in Figure 1.

**Table 2:** Prospective evaluation of static and functional balance in the scale of Berg, before and after the kinesitherapy (in points)

Parameters	At the beginning (n=56)	10^th^ day (n=56)	1^st^ month (n=56)	3rd month (n=56)
	X̄±S_D_	X̄±S_D_	X̄±S_D_	X̄±S_D_
**Static balance**				
Standing with collected feet (Berg 7)	1.7±0.7	3.6±0.4 ***	4.0±0.1 ***	3.9±0.1 ***
Standing with eyes closed (Berg 6)	1.4±0.5	3.3±0.5 ***	3.9±0.1 ***	3.9±0.2 ***
Standing tandem (Berg 13)	1.5±1.1	3.3±0.7 ***	3.6±0.4 ***	3.6±0.4 ***
Standing on one leg (Berg 14)	0.6±0.4	2.7±0.7 ***	3.4±0.5 ***	3.4±0.5 ***
**Functional balance**				
Getting up from seating to standing (Berg 1)	2.1±1.0	3.8±0.3 ***	4.0±0.1 ***	4.0±0.5 ***
Standing without support (Berg 2)	2.9±1.0	3.8±0.3 ***	4.0±0.2***	4.0±0.4***
Sitting without back support - feet on the floor (Berg 3)	3.9±0.9	3.8±0.4 ***	4.0±0.2 ***	4.0±0.4 ***
Sitting posture of (Berg 4)	1.7±0.5	3.7±0.4 ***	4.0±0.2 ***	4.0±0.5***
Moving forward from the seating of the chair (Berg 5)	2.4±0.6	3.9±0.2 ***	4.0±0.3 ***	4.0±0.4 ***
Reaching forward with outstretched hands (Berg 8)	1.7±0.6	3.7±0.4 ***	4.0±0.2 ***	4.0±0.5 ***
Lifting from the floor by standing (Berg 9)	1.8±1.1	3.5±0.6 ***	3.9±0.2 ***	3.8±0.3 ***
Rotate from standing (Berg 10)	1.6±0.6	3.7±0.4 ***	4.0±0.1 ***	4.0±0.1***
Rotate for 360 ° (Berg11)	1.8±0.8	3.5±0.6 ***	3.8±0.3 ***	3.8±0.3 ***
Of standing without support with hands- stepping on a block sequentially with both feet (Berg 12)	1.4±1.0	3.1±0.9 ***	3.6±0.4 ***	3.6±0.4 ***

X̄ ± SD – average value and standard deviation,

*** p <0.001 = significant difference compared with baseline values.

**Table 3:** Prospective evaluation of the total number of points on the Berg Balance Scale, before and after kinesitherapy (in points)

Parameters	At the beginning (n=56)	10th day (n=56)	1st month (n=56)	3rd month (n=56)
X̄±S_D_	X̄±S_D_	X̄±S_D_	X̄±S_D_
Total Points	26.85±9.02	49.92±5.67 ***	54.57±1.71 ***	54.32±2.07 ***

X̄ ± SD - averagevalue and standard deviation,

*** p <0.001 = significant difference compared with baseline values.

Patients were found to improve significantly in balance opportunities, according to the scale of Berg. Compared to baseline (initial) data there is a significant increase in the number of points in the measured indicators for functional and static balance (Table 2).

Such are the results of the points, according to the same test. The initial amount is 26.85, then a tendency to increase to 49.92 points can be noticed on day 10. In absolute terms, positive change is most pronounced during the 1st month (54.57), with a level of significance p <0.001 (Table 3 and Figure 1).

There is a significant relationship between static and dynamic balance, with established objective correlations between indicators of the Berg Balance Scale during treatment.

The results obtained from static equilibrium parameter for Berg 7, Berg 13 and Berg 14, i.e. the possibility to stand with feet put together, putting one foot in front of the other leg and to stand on one leg are positively correlated with the changes observed in the indicators of functional balance of Berg 9 and Berg 12 on lifting an object from the floor by standing and standing without support of the arms, stepping on a block with both feet in turns / in sequence.

The increased ability in patients to stand alone with feet put together is associated with the increased opportunity to raise the object from the floor in a standing position, as between the change of indicators a significant positive correlation is registered (r = 0.445, p = 001) on the 10th day. During the 1^st^ month, the results of Berg 7 are constant and correlation cannot be calculated. During the 3^rd^ month of the observation, the correlation was r = 0.556 and p = 0.000 and adopted a significance level of p <0.01.

Improving the ability to stand alone with feet put together associated with increasing the possibility of standing without support with hands, stepping on a block with both feet in turns/in sequence, as between the change of indicators a significant positive correlation is registered (r = 0.768, p = 0.000, with significance level p <0.01) on day 10 of the study. During the 3rd month, the correlation was r = 0.189, p = 164 and is assumed to be correlated with the level of significance p <0.05. As already noted the 1st month results of Berg 7 are constant and correlation can not be calculated.

The results obtained from parameter Berg 13 on increasing the opportunity to put one foot in front of the other foot, arms crossed over the chest associate with improving the ability to raise the object from the floor in a standing position, as between the change of indicators a significant positive correlation is registered (r = 0.422, p = 001, with a level of significance of p <0.01) on day 10. During the 1^st^ month the correlation was r = 0.403, p = 002, with a level of significance of p <0.01. During the 3^rd^ month of the observation, the correlation was r = 0.556 and p = 0.000 and adopted a significance level of p <0.01.

Also improving the ability to put one foot in front of the other foot, with arms crossed over the chest is associated with increasing the possibility of standing without support of the arms, stepping on a block with both feet in turns / in sequence, as between the change of indicators a significant positive correlation is registered (r = 0.684, p = 0.000, with a level of significance of p <0.01) on day 10 of the study, which was significant during the 1^st^ month (r = 0.673, p = 0.000, with a level of significance of p <0.01) and during the 3^rd^ month (r = 0.542, p = 0.000, with a level of significance of p <0.01).

Improving the ability to stand on one leg is associated with an increased opportunity to raise the object from the floor in a standing position, as between the change of indicators a significant positive correlation is registered (r = 0.457, p = 0.000, with a level of significance of p <0.01) on day 10 of the study. During the 1^st^ month the correlation was r = 0.258, p = 0.55, and is not considered to be in correlation with the level of significance p <0.05), and during the 3rd month again it is believed to correlate, as the positive correlation is (r = 0.469, p = 0.000, with a level of significance of p <0.01).

Patients with an increased ability to stand on one leg are associated with an increased possibility of standing without support of the arms, stepping on a block with both feet in turns / in sequence, as between the change of indicators a significant positive correlation is registered (r = 0.789, p = 0.000, with a level of significance of p <0.01) on day 10 of the study, which was significant during the1st month (r = 0.641, p = 0.000, with a level of significance of p <0.01) and during the 3^rd^ month (r = 0.613, p = 0.000, with a level of significance of p <0.01).

## Discussion

This study shows that the use of a specialized 10-day SKTM, which was later continued as adapted exercise program at home for a period of three months, significantly improved static and functional balance of patients with ischemic stroke during the follow-up period. The positive change is permanent, with the clear improvement in the 1st month of treatment, and then the effect is continued up to 3 months of the treatment.

The beneficial effects of the applied physical therapy, developed on the basis of neural stimulation therapy of Bobath (Neurodevelopmental Treatment - NDT), connect to the included exercises to move from occipital to standing position, normalizing the control of the body and increasing autonomy in changing the basic position. The movements used for lower limb, pelvis, consistency of response and motor response in m. quadriceps femoris muscle strengthen and improve patients’ balance. The performance of these targeted movements associated with a shift of the body, lead to an increase in the speed and accuracy of performance [2, 3].

Similar findings from other studies suggest that neurostimulation therapy of Bobath (Neurodevelopmental Treatment - NDT) has a positive effect on the balance control of symmetrical distribution of body weight on paretic and nonparetic side and overall balance, objectified through the scale of Berg [20].

Several studies have shown that static balance, including training and sitting posture during various activities is essential for patients after stroke [26]. Stable standing requires a combination of elements including muscle strength, suitable afferent (low) signals and the ability to integrate these signals in the own body’s scheme. It is not enough to recover only one of the elements and this may lead to explanation, for example, why the physiological recovery of muscle function of paretic leg (especially in the first 3 months after stroke) does not necessarily lead to improved support functions and balance [27]. Static balance positively affects the functional balance [2, 3]. For the balance, important role play the following: muscle activity with increased demands on postural control, voluntary movements of the body and increased response in practice [28, 29, 30].

Lasting positive effect of the application of SKTM can be explained by the continuous three-month exercise, which improves the speed of performing the activities and postural control. Besides duration, repetition, focus and variability in performance of motor tasks are fundamental principles of motor training observed in the presented methodology. Repetitive tasks relate to an active motor sequence performed repeatedly during training with practices seeking clear functional purpose [31]. However, this does not mean that each repetition should be identical to the previous one. Instead, a slight variation between repetitions is applied for better training [32].

The applied specialized kinesitherapeutic methodology, based on the principles of motor control, motor training and modern guidelines on neurostimulate therapy (neurodevelopmental treatment (NDT) continued later as exercise program at home, which significantly improves static and functional balance in patients with ischemic stroke in the chronic period and is with a supportive prolonged exposure.
